# Using neural networks to autonomously assess adequacy in intraoperative cholangiograms

**DOI:** 10.1007/s00464-024-10768-0

**Published:** 2024-04-01

**Authors:** Henry Badgery, Yuning Zhou, James Bailey, Peter Brotchie, Lynn Chong, Daniel Croagh, Mark Page, Catherine E. Davey, Matthew Read

**Affiliations:** 1 Department of Biomedical Engineering, The University of Melbourne, Parkville, Australia; 2 Department of Upper Gastrointestinal Surgery, St Vincent’s Hospital Melbourne, Melbourne, Australia; 3 Department of Surgery, The University of Melbourne, St Vincent’s Hospital, 41 Victoria Parade, Fitzroy, Melbourne, VIC 3065 Australia; 4 School of Computing and Information Systems, The University of Melbourne, Parkville, Australia; 5 Department of Radiology, St Vincent’s Hospital Melbourne, Melbourne, Australia; 6 Department of Surgery, Monash Health, Melbourne, Australia; 7 Graeme Clark Institute for Biomedical Engineering, The University of Melbourne, Melbourne, VIC Australia

**Keywords:** Convolutional neural network, Cholangiogram, Laparoscopic cholecystectomy, Artificial intelligence

## Abstract

**Background:**

Intraoperative cholangiography (IOC) is a contrast-enhanced X-ray acquired during laparoscopic cholecystectomy. IOC images the biliary tree whereby filling defects, anatomical anomalies and duct injuries can be identified. In Australia, IOC are performed in over 81% of cholecystectomies compared with 20 to 30% internationally (Welfare AIoHa in Australian Atlas of Healthcare Variation, 2017). In this study, we aim to train artificial intelligence (AI) algorithms to interpret anatomy and recognise abnormalities in IOC images. This has potential utility in (a) intraoperative safety mechanisms to limit the risk of missed ductal injury or stone, (b) surgical training and coaching, and (c) auditing of cholangiogram quality.

**Methodology:**

Semantic segmentation masks were applied to a dataset of 1000 cholangiograms with 10 classes. Classes corresponded to anatomy, filling defects and the cholangiogram catheter instrument. Segmentation masks were applied by a surgical trainee and reviewed by a radiologist. Two convolutional neural networks (CNNs), DeeplabV3+ and U-Net, were trained and validated using 900 (90%) labelled frames. Testing was conducted on 100 (10%) hold-out frames. CNN generated segmentation class masks were compared with ground truth segmentation masks to evaluate performance according to a pixel-wise comparison.

**Results:**

The trained CNNs recognised all classes.. U-Net and DeeplabV3+ achieved a mean F1 of 0.64 and 0.70 respectively in class segmentation, excluding the background class. The presence of individual classes was correctly recognised in over 80% of cases. Given the limited local dataset, these results provide proof of concept in the development of an accurate and clinically useful tool to aid in the interpretation and quality control of intraoperative cholangiograms.

**Conclusion:**

Our results demonstrate that a CNN can be trained to identify anatomical structures in IOC images. Future performance can be improved with the use of larger, more diverse training datasets. Implementation of this technology may provide cholangiogram quality control and improve intraoperative detection of ductal injuries or ductal injuries.

**Graphical abstract:**

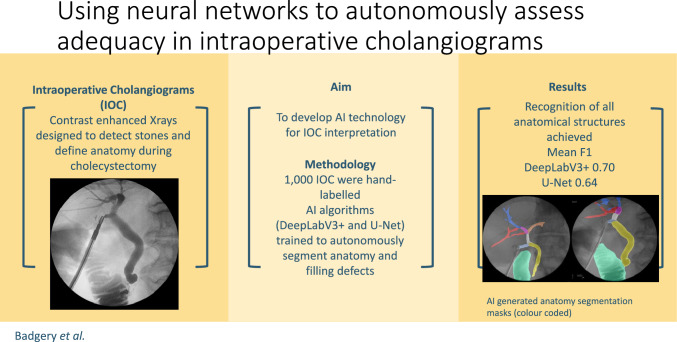

**Supplementary Information:**

The online version contains supplementary material available at 10.1007/s00464-024-10768-0.

Intraoperative cholangiography (IOC) is a contrast-enhanced X-ray study taken during laparoscopic cholecystectomy to display the biliary tree. IOC provides a dynamic method to image the biliary tree through radiographic visualisation of contrast flow through the biliary tree. IOC is used to detect the presence of stones in the common bile duct, define anatomy, and look for the presence of bile leak from a biliary tree injury. The use in Australia is comparatively high, being performed in over 80% of cholecystectomies, compared with rates internationally ranging from 20 to 30% [1–4]. Although IOC have not been demonstrated to reduce the rate of bile duct injury (BDI), they has clinical utility in being able to identify both filling defects and injuries to the biliary tree [5, 6].

An IOC is performed by injecting radio opaque contrast into the cystic duct while taking X-ray images using a portable X-ray machine (Fig. 1). Once the cystic duct is clearly identified and adequately dissected, a lateral incision is made, and the duct is cannulated. The cannula is used to infuse contrast into the duct under pressure to visualise the biliary tree. Interpretation of the cholangiogram involves recognition of five key features: (1) contrast flow into the duodenum; (2) distal filling of the common bile duct; (3) proximal filling of the three main hepatic ducts, i.e. the left hepatic duct (LHD), the right anterior hepatic duct (RAHD) and right posterior hepatic duct (RPHD); (4) the absence of filling defects in any ducts and (5) spiral valves visible within the cystic duct [7]. Recognition of filling defects intraoperatively provides an opportunity for early intervention or intervention at index operation. This can be achieved using a specialised camera and stone retrieval equipment via the cystic duct, or directly via choledochotomy (an incision into the bile duct). If surgical removal at index operation is not possible, early referral for endoscopic retrograde cholangiopancreatography (ERCP) is another option. Stents can also be placed at the time of operation to maintain duct patency. The immediate recognition of bile leak or BDI using IOC obviates the risk of delayed diagnosis, allowing for intervention at index operation or prompt transfer to a specialist centre for early intervention. Early recognition and management of bile leaks and BDI is key to improving outcomes [8]. Failure to achieve the five components of a cholangiogram should arouse suspicion for ductal injury or a retained stone or stricture. Furthermore, any technical issues should be rectified to ensure adequacy of the cholangiogram.

**Fig. 1:**
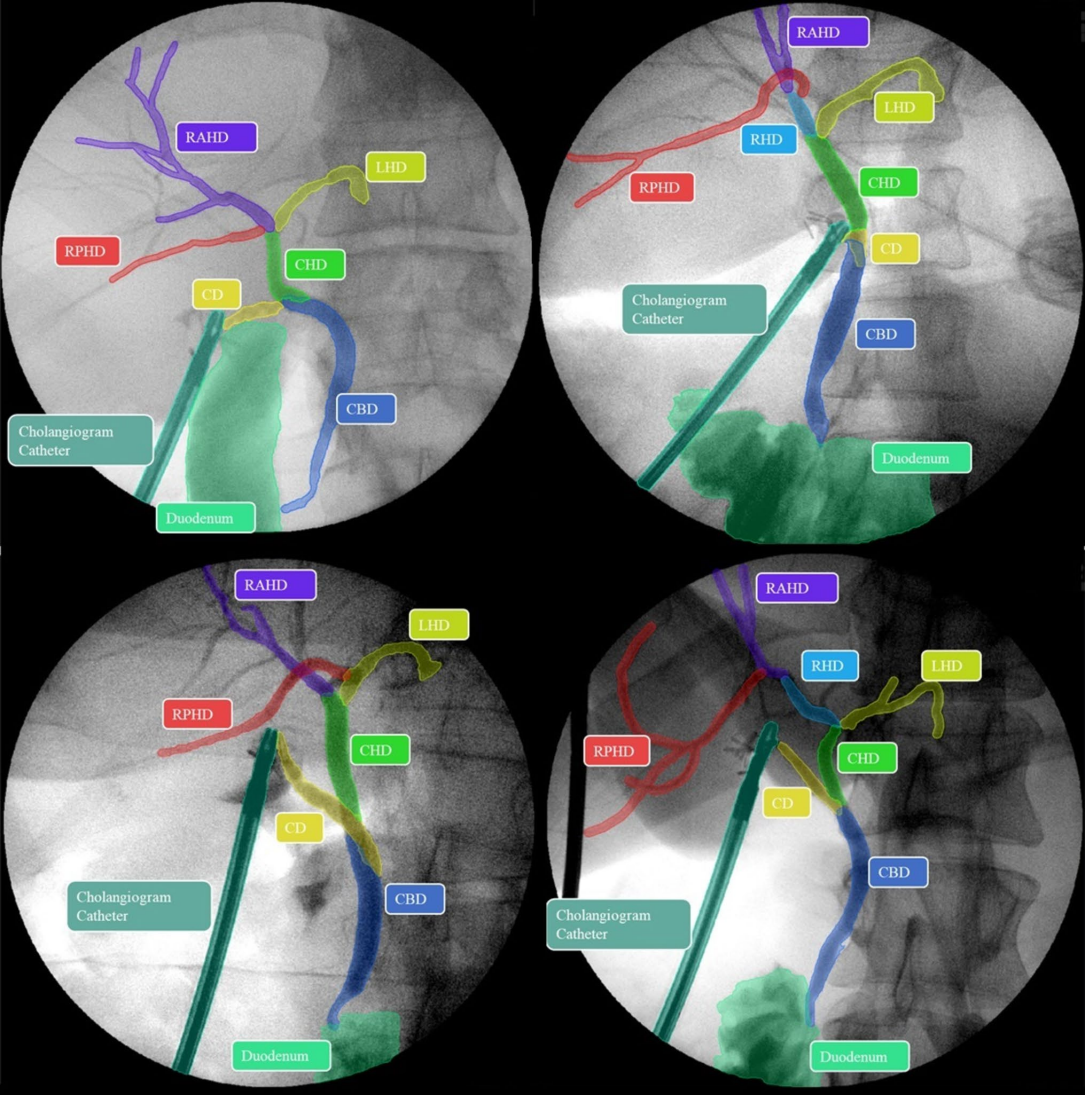
Examples of human applied segmentation masks applied to intraoperative cholangiograms (IOC). Masks correspond to labels listed in Table 1: *CBD* common bile duct, *CHD* common hepatic duct, *CD* cystic duct, *LHD* left hepatic duct, *RAHD* right anterior hepatic duct, *RHD* right hepatic duct, *RPHD* right posterior hepatic duct

Artificial intelligence (AI) is the use of computers or machines to to perform tasks that typically require human intelligence. Investment of AI in medical and surgical applications has surged in recent years, in part owing to access to improved computing power and data collection [9]. AI-based visual tasks and applications have been developed across a wide array of medical fields including radiology, surgery, endoscopy and pathology. Convolutional neural networks (CNNs) are a specific type of machine learning algorithm modelled on the structure and function of the biological neural system [10]. CNNs are effective in computer vision tasks such as classification, detection, or segmentation of structures in medical images. This technology has broad potential; in the context of IOCs, it can be used to generate an AI-powered checklist to ensure that the key cholangiogram features have been adequately demonstrated and no abnormalities are overlooked, serving as a safety checkpoint to avoid a missed injury or retained stone. A segmented visual image can also be used as an improved form of documentation detailing a satisfactorily completed cholangiogram. Furthermore, this technology could serve as a training adjunct to surgical trainees and help from the basis for augmented reality environments were AI-defined anatomical maps can help guide surgeons intraoperatively or provide opportunities for preoperative modelling and simulation.

In this project, we aim to train CNNs to accurately recognise and segment key anatomical structures in IOC. This is the first published work outlining the use of AI and computer vision in IOC.

## Methods

Ethics approval for this study was obtained from the St Vincent’s Hospital, Melbourne Human Research Ethics Committee (St Vincent’s HREC reference HREC/67934/SVHM-2020-235987, protocol amendment V2 January 2022) with governance approval obtained for peripheral contributing sites.

### Data collection

IOC was retrospectively obtained from three tertiary hospitals through the imaging archiving system with cases matched through the hospital coding systems. After retrieval of cholangiograms, images were manually selected based on the following criteria: (a) minimum of one and maximum of two frames per patient; (b) best quality frame(s) selected per patient; and (c) if the entire biliary tree is not visualised in a single frame, two included frames in combination should demonstrate the entire biliary tree. A maximum of two images per patient was implemented to limit imbalance within the dataset. The best quality frame(s) were selected for each patient based on visual assessment. Frames were assessed based on clear representation of all structures with minimal movement artefact. Frames with a presence of other obstructing structures such as instruments, cables, bony structures or leaked contrast were excluded. Metadata were stripped from all files and images were converted and stored securely as png files.

### Dataset preparation

The dataset in its entirety was reviewed by a surgical trainee to ensure consistency. A purpose-written script was used to automatically deidentify images including removal of patient information visible on the Xray. A labelling protocol was written to encapsulate the key anatomical structures, filling defects and the cholangiogram catheter (Table 1). A total of 10 classes were included plus a background class for unlabelled pixels. Segmentation masks were applied by a general surgery trainee with previous labelling experience using Darwin V7 platform (V7 Labs, 2020) [11]. 1,000 frames taken from 586 patients were ultimately labelled and included in the dataset (Table 2). Codes were assigned to each cholangiogram, including patient-specific codes so that cholangiograms obtained from the same patient for the same procedure could be identified. Patients in the testing dataset were kept distinct from the training dataset to prevent data bleeding between the different sets.

**Table 1:**
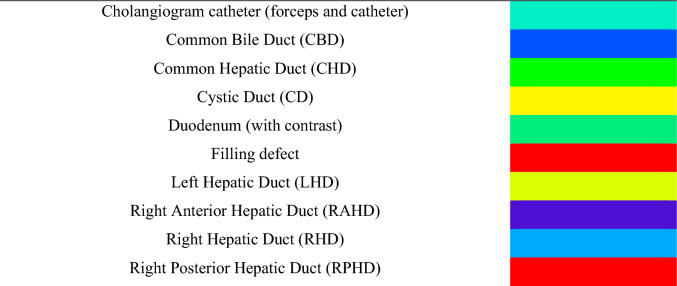
Intraoperative cholangiogram (IOC) segmentation labels with corresponding colours as applied by Darwin V7

**Table 2:** Dataset summary

Dataset	Frames	Patients
Training	720	420
Validation	180	107
Testing	100	59
Total	1000	586

The testing dataset of 100 frames (10%) was reviewed by an experienced abdominal radiologist to ensure that all anatomical labels were accurate. Minor adjustments were made where necessary. The test set masks were compared pre- and post- review adjustments, yielding a mean F1 score of 0.99 across the testing dataset. The remaining 900 frames (90%) were then split into a training dataset of 720 frames (80%) and a validation dataset of 180 frames (20%).

### Dataset augmentation and network selection training

Our dataset underwent augmentation to increase data volume. Augmentation techniques included horizontal flipping and random colour jittering within a specified range (brightness 0.25, contrast 0.25, saturation 0.25 and hue 0.0). In addition, random rotation was applied between -30 degrees and 30 degrees. Rotation was confined to this range as this reflects the real-world variability in cholangiogram X-ray orientation. For computational efficiency, frame resolution was downsized to 300 × 300 pixels. To mitigate the problem of dataset class and pixel imbalance, specific weightings were applied during training to each class. These weightings were inversely proportional to class prevalence. Two CNNs, namely DeeplabV3 +  [12] with a ResNet101 [13] backbone (Supplementary Fig. 1) and U-Net [14] (Supplementary Fig. 2), were selected and trained separately using the same labelled and augmented dataset. Comparative analysis of the two networks was performed following training. DeeplabV3 + is a powerful CNN that performs well in computer vision tasks [12]. It was chosen after preliminary success in early experiments using a pilot dataset of 70 labelled cholangiogram frames. The second network trained was U-Net, a CNN that was specifically designed for biomedical image segmentation [14]. Deeplabv3 + was run with a ResNet101 backbone while U-Net was run without a backbone. DeeplabV3 + with ResNet101 backbone had almost 9 times the parameters of the U-Net architecture and conducted five times more multiply-accumulate computations for each feed-forward training iteration [13]. Training experiments were conducted on four NVIDIA A100 graphics processing units (NVIDIA, Santa Clara, California, USA) with PyTorch implementation on the Spartan high-powered computer housed at the University of Melbourne. Hyperparameters were optimised using the validation dataset. Both models were trained using AdamW as the optimizer for 100 epochs, with 10 warm up epochs [15, 16]. The batch size was set to 64, the initial learning rate 0.005, and weight decay 0.01. Given the relatively small testing dataset size, K-fold cross-validation was conducted (K = 5).

### Evaluation metrics

**Fig. 2:**
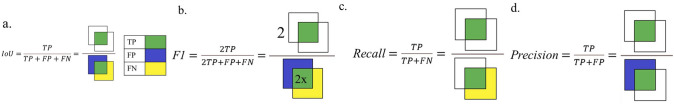
Equation and diagram for evaluation metrics. **a** Intersection-over-Union (IoU); **b** F1/Dice Coefficient; **c** Recall; **d** Precision

After training, network accuracy was evaluated by comparing the network prediction with the ground truth of human annotations, as demonstrated using common evaluation metrics. These metrics included intersection over union (IoU), F1 coefficient, recall and precision as well as true positive (TP), false positive (FP), true negative (TN) and false negative [17] (FN) (Fig. 2). TP, FP, TN and FN refer to each pixel prediction and its concordance with the ground truth pixel-wise label. In addition to the described evaluation metrics, correct recognition of the presence of an object was also determined where the network generated segmentation mask overlapped with the ground truth segmentation mask. This was calculated by determining any degree of accurate overlap between ground truth and the prediction segmentation mask, without consideration of the pixel-wise segmentation accuracy.

IoU, otherwise known as the Jaccard similarity coefficient, is one of the most commonly used metrics for computer vision evaluation [18]. It is the area of common overlap between the ground truth and the network prediction, ranging from 0 to 1 (0% to 100%) with 0 being no overlap and 100 being perfect concordance [19]. In object detection applications, IoU > 0.5 is considered a good score and represents adequate localisation, though the required precision varies depending upon network application [20, 21].

The F1 coefficient, otherwise known as the Dice similarity coefficient or the Sorensen-Dice index, is similar to IoU in that it measures overlap between ground truth and prediction. It differs in that it represents the harmonic mean between sensitivity and precision. IoU penalises over and under-segmentation more than the F1 coefficient [19, 21]. Similar to IoU, the F1 range is from 0 (no concordance) to 1 (perfect concordance) with a value greater than 0.5 considered good, depending upon the application [21].

Recall, otherwise known as the sensitivity or true positive rate, demonstrates the rate of correctly attributed pixels by calculating the ratio between the network prediction attribution of positive pixels and all pixels attributed to that class. Recall is particularly useful in medical diagnosis applications where false-negative rate is penalised and correct attribution of pixels is rewarded [17].

Class precision is calculated as the ratio between correct class pixel predictions and all pixels assigned to the relevant class. False-positive predictions are penalised in precision metrics [17].

## Results

Both the DeeplabV3 + and U-Net networks performed well, achieving a mean F1 coefficient of 0.70 and 0.64, respectively, excluding the background class (Table 3). There was a degree of imbalance in classes in terms of incidence of structure representation as well as proportion of pixels attributable to each structure in the dataset. Larger and more distal structures (e.g. CBD, duodenum, CHD) had greater representation than the finer and more proximal higher order ductal structures (e.g., hepatic ducts) (Table 3). The cholangiogram catheter, CBD, CHD, CD, duodenum, LHD and RAHD were correctly identified without perfect segmentation in over 80% of the testing dataset. Filling defects were present in 21% of frames in the testing dataset (21/100). While the pixel-wise accuracy of filling defect segmentation was poor in both networks (DeeplabV3 + F1 0.34, UNet F1 0.24), the presence or absence of filling defects was correctly characterised in 89% of cases for the whole testing dataset (89/100) and in 66% of cases (14/25) in cholangiograms where a filling defect was present. Of the filling defect errors, 37% (4/11) represented a network detection where a filling defect was not present, and 63% represented failure to recognise a filling defect where one was present. This is reflected by a high false negative rate (78% in DeeplabV3 +). K-fold cross validation performed on DeeplabV3 + demonstrated a mean IoU of 0.61, (SD = 0.0045), while the UNet mean IoU was 0.52 (SD = 0.017), suggesting superior performance and greater stability of DeeplabV3 + when trained and tested across different dataset subsets.

**Table 3:**
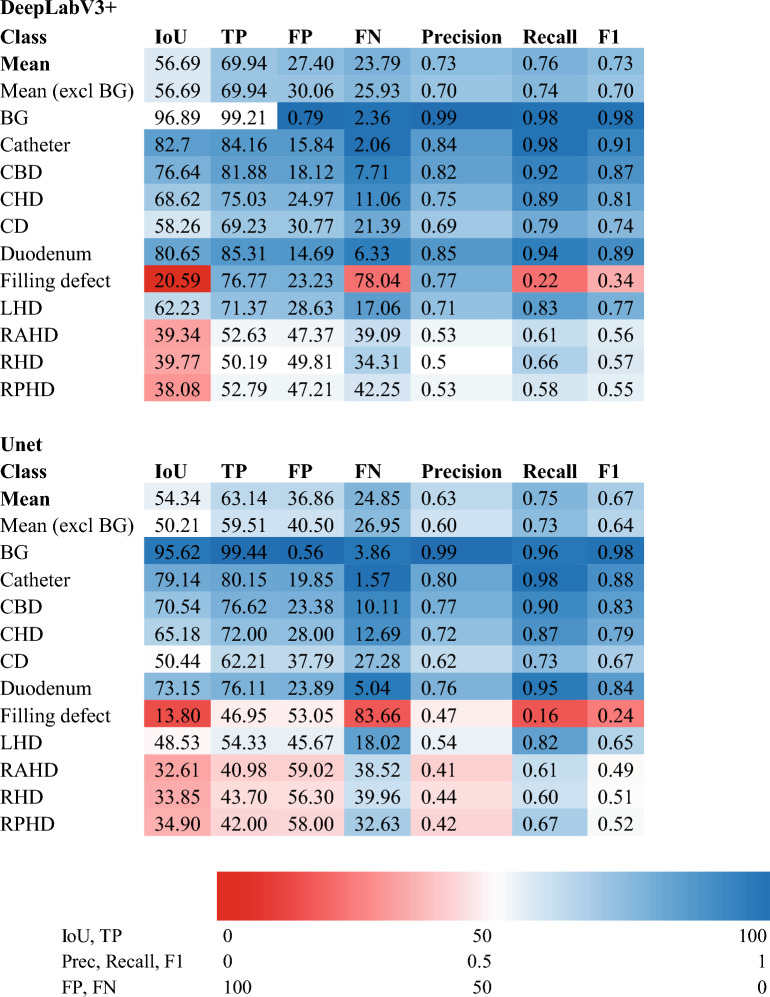
Results of trained Deeplab V3+ convolutional neural network (CNN) (above) and U-Net (below) networks segmenting image frames using an unseen, hold-out test dataset

*IoU* intersection over union, *TP* true positive, *FP* false positive, *FN* false negative, *BG* Background

**Fig. 3:**
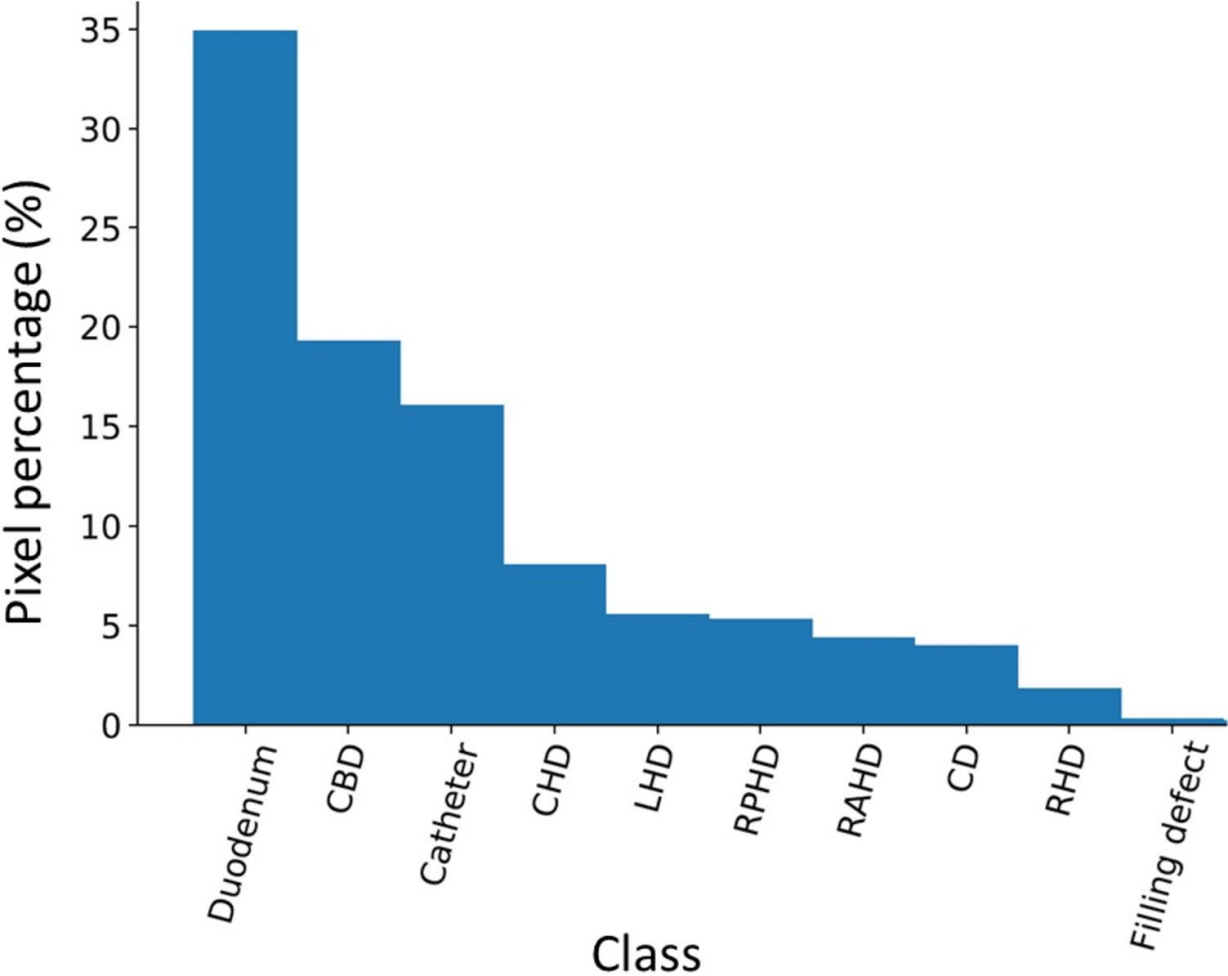
Class frequency: The pixel class distribution expressed as a percentage of all pixels attributable to each class after exclusion of background

Class-specific performance was strong on most structures. Both networks achieved an IoU of over 0.5 on all anatomical structures, except for the filling defects and the right hepatic duct group (RHD, RAHD and RPHD) (Fig. 3). F1 generated by DeeplabV3 + for CBD, CHD, CD and LHD were > 0.7 whereas F1 scores for the right ducts were between 0.5 and 0.6. F1 scores were higher than IoU scores in all classes. The networks generally performed better on classes with a higher pixel representation (eg. Duodenum, CBD, catheter, CHD). Higher order structures or structures with a lower pixel representation, such as the hepatic duct branches and filling defects were less accurately segmented. DeepLabV3 + outperformed U-Net on nearly all classes and evaluation metrics with the exception being the recall for the RPHD.

**Fig. 4:**
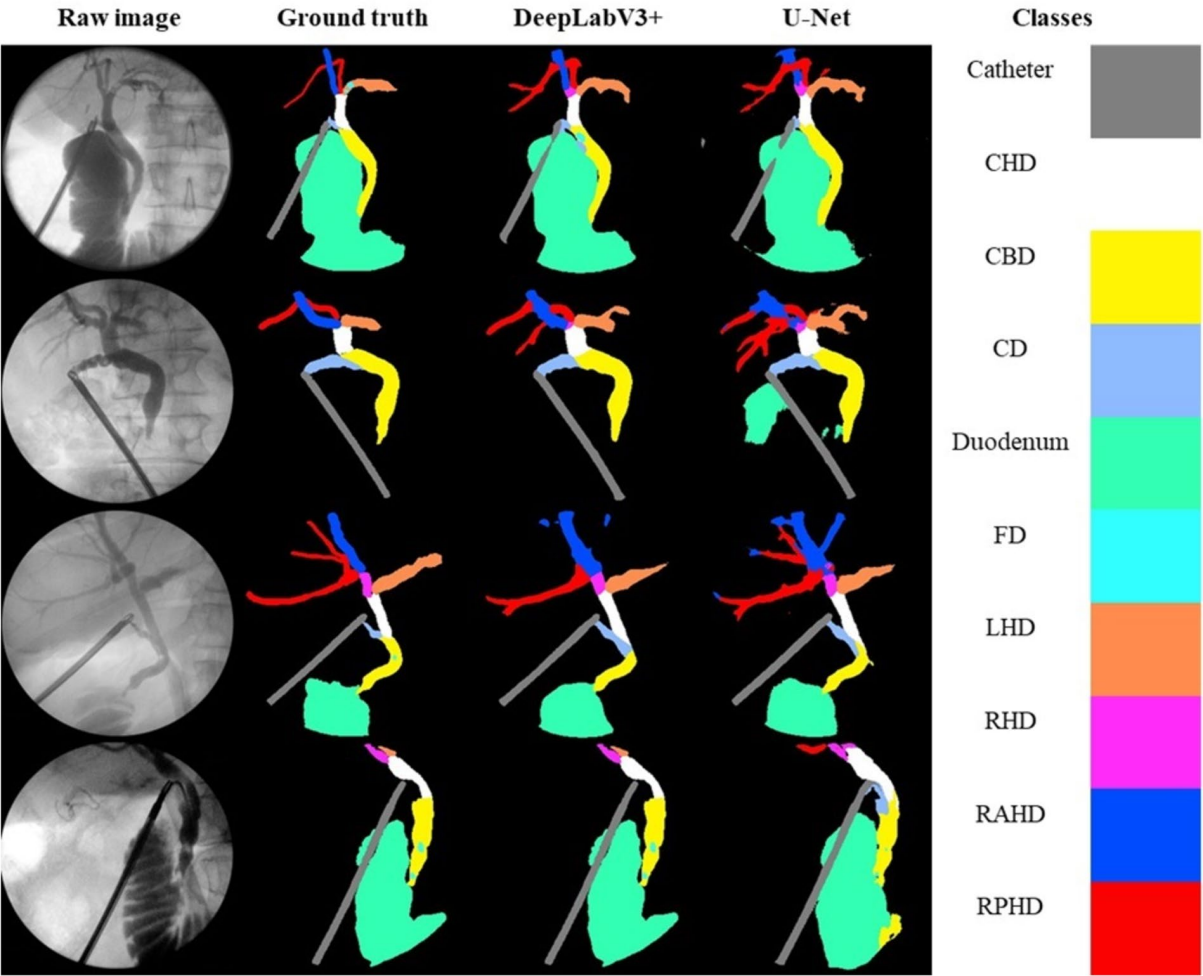
Examples of convolutional neural network (CNN) generated masks compared with ground truth labels with accompanying colour legend from hold-out test dataset. Figure depicts original cholangiogram image and human labelled ground truth segmentation masks with DeepLabV3 + and U-Net prediction segmentation masks for side-by-side comparison. Colour legend altered from Fig. 1 and Table 1 for visual clarity. *CHD* common hepatic duct, *CBD* common bile duct, *CD* cystic duct, *FD* filling defect, *LHD* left hepatic duct, *RHD* right hepatic duct, *RAHD* right anterior hepatic duct, *RPHD* right posterior hepatic duct

**Fig. 5:**
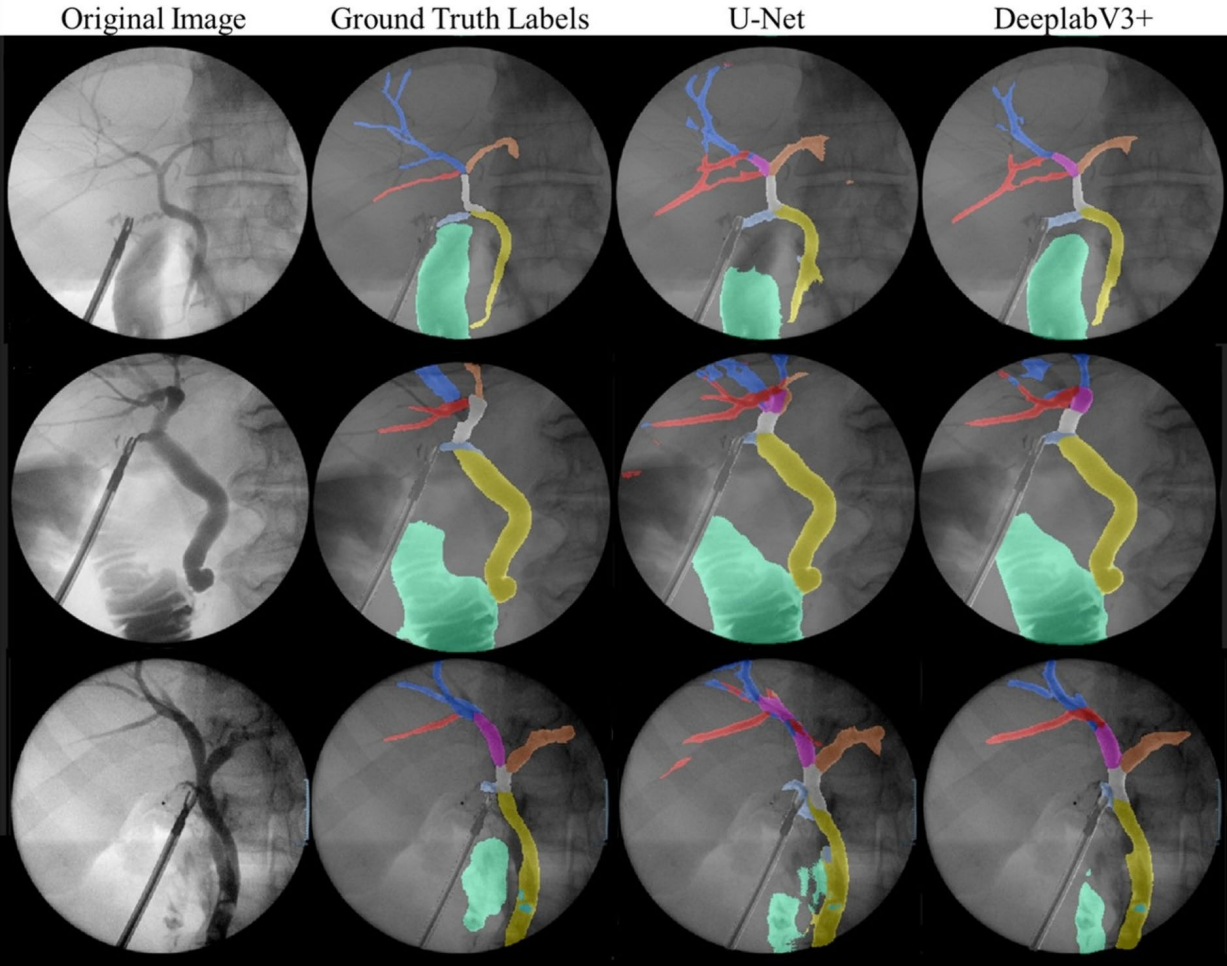
Comparison of ground truth composite mask overlay with DeeplabV3 + and U-Net composite mask overlay

Direct visual comparison between network prediction on the 100frame testing dataset reveal superior performance by DeeplabV3 + . This is demonstrated on the colour coded segmentation masks (Fig. 4) as well as composite images of segmentation masks superimpose upon original images. (Fig. 5). DeepLabV3 + appears to make fewer mistakes globally (Fig. 5). In some instances, peripheral minor hepatic duct branches that were left unlabelled in the ground truth dataset were accurately labelled by the predictive networks (Fig. 5).

## Discussion

In this feasibility study, we have demonstrated a novel application of computer vision in laparoscopic cholecystectomy surgery. We have outlined our dataset of cholangiogram comprehensively segmented into ten classes. Using this locally acquired pilot dataset of labelled cholangiograms, we have achieved a high degree of accuracy in anatomical segmentation using two CNN models with anatomical structures correctly recognised in over 80% of cases and filling defects correctly characterised in 73% of cases. These results provide proof of concept in the development of an accurate and clinically useful tool to aid in the interpretation and quality control of intraoperative cholangiograms.

To our knowledge, this is the only existing dataset of semantic segmentation labelled cholangiograms. The use of CNNs to autonomously segment intraoperative cholangiograms is novel and has many potential applications. Despite the modest dataset size, we have trained two networks capable of identifying key structures. In its current state, the network can be implemented in an autonomous surgical checklist that identifies key cholangiogram features, including contrast flow to the duodenum, the presence of all three hepatic ducts and the visualisation of cystic duct draining to CBD with proximal filling.

One major strength of our trained networks is the capacity to recognise anatomy despite inconsistencies in cholangiogram acquisition and projection. Unlike other imaging modalities, such as frontal X-ray or axial CT that utilise consistent and protocolised projections, cholangiograms are taken with random oblique projections, depending upon patient and operating table position. This adds an additional layer of complexity in autonomous structure recognition using neural networks. Despite the inconsistency in projection, our trained networks were still able to achieve a good result. In addition to projection variability, different X-ray machines were used across multiple health services leading to heterogeneity in cholangiogram appearance. Despite the cholangiogram variability stemming from the use of different machines, our trained networks performed well, highlighting the robustness of the algorithms.

The detection of fillings defects and stones is an important function of cholangiogram. Accurate detection by a network is therefore an important capability to justify clinical implementation. While we achieved correct characterisation of filling defects in most cases, the network failed in several instances. The failure to consistently recognise the presence of filling defects likely stems from several factors. Stones are detected in a small minority of cholangiograms with reported rates ranging from 5 to 20% [22–25]. In our dataset, there were 227 frames containing fillings defects and a total of 363 instances representing just over a quarter of total frames. This rate of filling defects is higher than the general population cholangiogram rate for stones. It is also important to note that cholangiogram filling defects are small and make up a disproportionately low fraction of the segmented area. Furthermore, the appearance of stones and fillings defects on cholangiogram is not consistent. Stones can appear as an absence of downstream contrast flow or a single or multiple rounded filling defects. Another issue with filling defect detection is their similarity in appearance to the background given that they represent a radiolucent absence of contrast. It can be difficult to distinguish filling defects from other cholangiogram features on single static images. The operating surgeon obtains important information from the dynamic images as the cholangiogram is being taken. The subtle signs suggestive of a stone are better appreciated on these dynamic images. These include the pattern of movement of a filling defect distinguishing it from an air bubble and the dynamic response of a filling defect to contrast flow. Improved recognition of stones could be achieved in several ways. The addition of more cholangiogram images positive for stones may improve results. Another method might be to explicitly classify features that are predictive of stone presence. These features might include an absence of downstream flow and dilatation of the cystic or common bile duct. Incorporation of these features in the training pipeline might improve downstream detection of stones.

Within individual cholangiograms there is a degree of ambiguity. Cholangiograms are 2-dimensional representations of 3-dimensional structures. The true anatomy, particularly in the hepatic ducts, can be misinterpreted due to X-ray projection and superimposition of ductal structure, bony structures, or instruments. Our network utilised exclusive classes, whereby each pixel could only be attributed to a single class. The use of non-exclusive classes, whereby a pixel could be attributed to multiple classes may further improve the accuracy and representation of the biliary tree by allowing for and identifying overlapping structures.

The assessment and evaluation of network performance warrants interrogation. All evaluation metrics employed in this study are a comparison of the network prediction with the ground truth, as determined by surgical trainee labellers. There are limitations in this approach. The ground truth labels contain a degree of subjectivity. The objectivity and truth of the training and testing datasets, therefore, are imperfect. The specific evaluation metric chosen to reflect performance must take into consideration the intended function or application of the network. In our trained networks, smaller higher order ducts tend to be less well segmented than larger calibre distal ducts. The segmentation prediction may not comprehensively detect all higher order hepatic duct branches however does succeed in recognising that the three main hepatic duct branches (LHD, RPHD, RAHD) have been adequately demonstrated. Failure to accurately segment all higher order branches will be penalised by the mathematical evaluation metrics but does not limit the clinical utility. This important point is demonstrated in Fig. 5. In the ground truth labels, higher order branches of the RPSD were not labelled however these were accurately segmented by U-Net and DeeplabV3 + . The mathematical evaluation metrics will categorise the predicted segmentation of these higher order branches as false positives given the discordance with the ground truth. While the accurate recognition of these higher order branches does not alter the clinical utility of the network, the performance is penalised. Similarly, accurate segmentation of all duodenal contrast by a network is not necessary where the function is the binary detection of the presence or absence of contrast flow into the duodenum. In such applications, a lower IoU can be tolerated provided the false positive rate is also low. Conversely, applications demanding a higher degree of accuracy such as measurement of the CBD diameter or cystic duct length demand more accurate segmentation ability of these structures. The intended output or clinical application of a network therefore must be considered when choosing appropriate evaluation metrics and interpretating prediction results.

Visual inspection of the prediction masks demonstrates superior performance by DeeplabV3 + as also reflected in the calculated evaluation metrics. Important structures, on visual assessment, are more accurately and consistently segmented. There is also comparatively less misrecognition. The modestly superior global performance of the DeepLabV3 + may be attributed to both the model size and its use of atrous separable convolutions that improve computational efficiency and reduce complexity [12]. Using a large backbone allows preservation of the understanding of the relative relationship and location of the structures and their adjacent objects. In Addition, atrous convolution allows the model decoder to receive larger contextual information from the previous feature maps while retaining spatial resolution. This can help the model to attain precise localization ability for the structures’ location. However, we argue that a larger model is not necessary to achieve better performance. We observe that although the model architecture and size are very different, the performance between the two networks is competitive. In some individual classes DeepLabV3 + performs worse than U-Net. U-Net identifies the subtle structure boundaries more precisely and has fewer FN, which suggest that the model is less likely to misattribute one structure to another. It is also superior at identifying small and underrepresented structures like filling defects. Using a heavy backbone like ResNet101 in DeeplabV3 + may also require more data to converge the larger model. In the biomedical and surgical context, adopting a large model on small datasets may lead to poorer performance, especially in identifying the under-represented classes with overfitting more likely to become a problem [26].

There were several limitations in our methodology. All cholangiograms in the dataset were labelled or finalised by one surgical trainee. The gold standard for semantic segmentation would be to have multiple trained experienced labellers segment each image and then use a concordance map or heatmap to determine the final semantic segmentation masks. The testing dataset was reviewed by a consultant radiologist and then adjusted accordingly by the surgical registrar to ensure ground truth was as accurate as possible. A more robust labelling and validation pathway would be justified in future projects. Another limitation was the local source of the cholangiograms. Our dataset was collected from three hospitals with cholangiograms conducted by a small group of surgeons. The CNN trained from a local dataset may not be as effective or applicable to cholangiograms performed internationally where local protocol and equipment may differ. Our network was trained on still images. However, intraoperative cholangiograms are dynamic investigations where information can be gained through tactile feedback from the pressure in the contrast syringe, observation of the rate of contrast flow through ducts and the movement of fillings defects to help distinguish stones from air bubbles. In some cases, static images are obtained to the satisfaction of the surgeon, but the corresponding stills are not captured for storage which may impact the apparent accuracy of the networks when considering the retrospective training datasets. Training from still images is therefore limiting. Future work should include dynamic or prospective real-time cholangiogram videos that can provide more information. An additional limitation relates to the chosen class list (Table 1). The labels defined for the hepatic ducts only took into account those demonstrated in the most common anatomical configurations. Accessory hepatic ducts that are seen in rare anatomically aberrant cases were not explicitly labelled. Furthermore, other structures commonly seen such as the pancreatic duct, or the gallbladder in the case of retrograde contrast flow, were not explicitly labelled. Therefore, in cholangiograms where these structures are demonstrated, our network will not be able to segment and label them accurately. Given these structures are uncommon, a substantially larger training dataset would be required to accurately incorporate these labels into a network.

There is great potential for the future direction of this work. With a local pilot dataset, we have achieved strong results while identifying clear strategies where these results can be improved. As discussed, larger more balanced datasets are needed to improve the performance of this CNN. Prospective evaluation of the network will demonstrate and better elucidate the clinical potential. The development of software aimed at autonomously checking that all five cholangiogram features have been satisfied is an important future aim that has real translational potential. Furthermore, international, or multi-site dataset collaborations will improve the performance and generalisability of this network.

## Conclusion

In this feasibility study, we have demonstrated the use of CNN based methods to detect and segment key anatomical structures on intraoperative cholangiogram. This work provides a platform for the development of ML based software for use in intraoperative cholangiograms. This software can serve as an autonomous safety checklist as well as a training and education tool. This may have utility in (a) surgical training and coaching, (b) auditing of cholangiogram quality, and (c) intraoperative safety mechanisms minimising the risk of missed ductal injury or stone. While the ability to detect and segment filling defects was average, we have discussed and identified methods of improving this performance that can be implemented in future work.

### Supplementary Information

Below is the link to the electronic supplementary material.

Supplementary file1 (JPG 39 KB)—DeepLabV3+ architecture [12]

Supplementary file2 (JPG 31 KB)—U-Net architecture [14]
