# Supplementary figures and images for: Using neural networks to autonomously assess adequacy in intraoperative cholangiograms
Source: Surg Endosc. 2024 Apr 1;38(5):2734–45. doi: 10.1007/s00464-024-10768-0 (PMC11078812; doi:10.1007/s00464-024-10768-0)

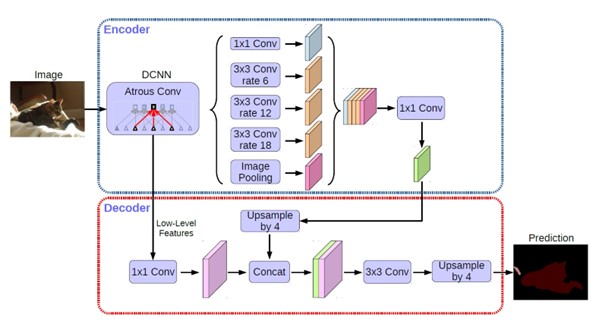

Supplement: Supplementary file 1 — Supplementary file1 (JPG 39 KB)—DeepLabV3+ architecture [12] [file 464_2024_10768_MOESM1_ESM.jpg]

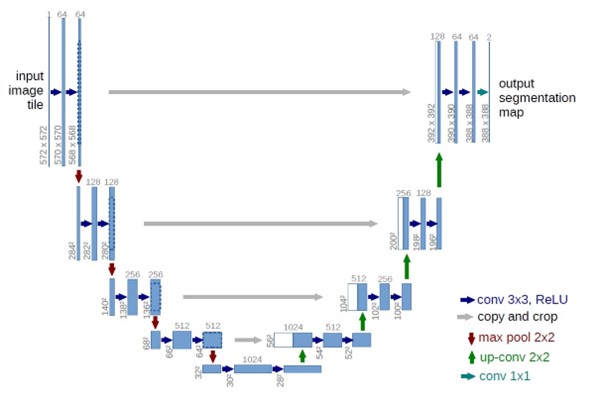

Supplement: Supplementary file 2 — Supplementary file2 (JPG 31 KB)—U-Net architecture [14] [file 464_2024_10768_MOESM2_ESM.jpg]
